# High-resolution crystal structure of spin labelled (T21R1) azurin from *Pseudomonas aeruginosa:* a challenging structural benchmark for *in silico* spin labelling algorithms

**DOI:** 10.1186/1472-6807-14-16

**Published:** 2014-05-29

**Authors:** Nicole Florin, Olav Schiemann, Gregor Hagelueken

**Affiliations:** 1 Institute for Physical and Theoretical Chemistry, University of Bonn, Wegelerstr. 12, Bonn, NRW 53115, Germany

## Abstract

**Background:**

EPR-based distance measurements between spin labels in proteins have become a valuable tool in structural biology. The direct translation of the experimental distances into structural information is however often impaired by the intrinsic flexibility of the spin labelled side chains. Different algorithms exist that predict the approximate conformation of the spin label either by using pre-computed rotamer libraries of the labelled side chain (rotamer approach) or by simply determining its accessible volume (accessible volume approach). Surprisingly, comparisons with many experimental distances have shown that both approaches deliver the same distance prediction accuracy of about 3 Å.

**Results:**

Here, instead of comparing predicted and experimental distances, we test the ability of both approaches to predict the actual conformations of spin labels found in a new high-resolution crystal structure of spin labelled azurin (T21R1). Inside the crystal, the label is found in two very different environments which serve as a challenging test for the *in silico* approaches.

**Conclusions:**

Our results illustrate why simple and more sophisticated programs lead to the same prediciton error. Thus, a more precise treatment of the complete environment of the label and also its interactions with the environment will be needed to increase the accuracy of *in silico* spin labelling algorithms.

## Background

The structural characterization of proteins by pulsed EPR methods such as pulsed electron electron double resonance (PELDOR, also known as DEER) has become increasingly popular in recent years [1-3]. A prerequisite for the PELDOR experiment is the presence of at least two paramagnetic centres in the protein. Since most proteins are diamagnetic, spin labels such as MTSSL [4] are routinely attached to the surface of proteins via site-directed spin labelling techniques [5-7]. The PELDOR experiment measures distances between such spin labels. The obtained distance information can then be used to analyse conformational changes e.g. of membrane proteins [8-10] or to reconstruct macromolecular complexes [11,12]. Although spin labels, such as MTSSL, are small compared to FRET labels (MTSSL has roughly the size of an arginine residue), they still act as a flexible link between the protein and the spin-centre itself (usually the nitroxide group of MTSSL). Thus, the problem arises, that on the one hand, the PELDOR experiment delivers an accurate distance between the two spin centres, but on the other hand, the exact position of the distance vector with respect to the protein is unknown. Many studies have investigated this problem. For example, the crystallographic analysis of multiple spin labelled T4-lysozyme mutants provided insights into the interactions of spin labels with proteins and preferred rotameric states of the labels [13-16]. The problem also led to the development of *in silico* spin labelling programs, which aim to predict the conformation of the spin label in the local environment of the attachment site [17-21]. These programs attach a model of the spin label to a macromolecular structure and generate an ensemble of possible rotamers of the label. The programs either allow all possible rotamers (e.g. for MTSSL: the five dihedral angles (χ1- χ5) are randomly set, [20]) or the angles are set based on calculated rotamer libraries and/or crystal structures [18,19,22]. With each method, distances between the generated ensembles can be measured and compared to experimental distances. Surprisingly, extensive benchmarks have revealed that independent of the approach, the average error of the predicted distances is around 3 Å, [20,22,23]. This indicates that current *in silico* spin labelling programs do not accurately enough model the complex interactions between a spin-label and its molecular environment. It is therefore important to gain further experimental insight into label-environment interactions. This will help to further extend available rotamer libraries with experimental rotamer data and provide clues concerning possible improvements of *in silico* spin labelling algorithms.

We present here the high-resolution (1.5 Å) structure of azurin from *Pseudomonas aeruginosa* PAO1 spin labelled with MTSSL at position T21. Due to the crystal-packing environment in the triclinic crystals, the label is observed in two different but fully ordered states. The excellent quality of the electron density allows precise measurements of all five dihedral angles in both conformations. Whereas one of the conformations fits to calculated rotamer libraries [18] the second conformation does not, illustrating how the environment of the label can force it to adopt a conformation which is less favourable for the free label. The implications for *in silico* spin labelling are discussed. Further, we present a newly developed, affinity chromatography based purification and spin labelling protocol for azurin.

## Results

### Protein production, spin labelling, crystallisation and structure solution

The classic purification protocol for azurin describes the isolation of recombinant azurin from the periplasmic fraction of *E. coli*[24]. Although this reportedly works well, we aimed to use affinity chromatography methods to streamline the production of azurin and to be able to use “on-column” spin labelling. To achieve this, the coding sequence for the mature azurin protein (excluding the coding sequence for the signal sequence) was cloned into the pHisGSTTEV vector. The T21C mutant was introduced by PCR techniques [25]. The protein was then overexpressed in *E. coli*, bound to the Ni^2+^ affinity resin, washed and spin labelled during the elution step. The GST tag was cleaved off with TEV protease and removed by gel filtration. Using this method, ~1 mg of pure, spin labelled azurin was obtained from a 1 l bacterial culture. Note that this yield is lower than the yield reported for the conventional protocol [24]. However, in our hands both methods yielded very similar amounts of pure azurin. The spin labelled protein (10 mg/ml) was crystallized at room temperature using a commercial crystallisation screen (JCSG+) and several initial hit conditions were obtained. The components of these conditions were combined in a stochastic optimisation screen. After several days, plate shaped, blue azurin crystals (~100 μm) appeared. A 200° diffraction dataset was collected at BESSYII (BL14.1). The data could only be processed and scaled in space group P1. The structure was then solved by molecular replacement using the structure of zinc bound azurin as search model (PDB-ID: 1E67 [26]) and refined until R/R_free_ converged at 18.8/21.4. Data collection and refinement statistics are summarized in Table 1. The refined structure was analysed with LABELIT to confirm that no higher crystallographic symmetry than P1 is present [27].

**Table 1:** Data collection and refinement statistics

**Data collection**	**Azurin T21R**_**1**_
PDB-ID	4BWW
Space group	P1
Unit cell (Å, °)	37.0, 53.7, 73.2, 74.4, 89.3, 83.4
Matthews coefficient (Å^3^ Da^-1^)	2.41
Solvent content (%)	49.0
Molecules per ASU	4
Wavelength (nm)	0.918409
Beamline	BL14.1, BESSYII
Resolution range (Å)	51.3-1.48
Total observations	155295
Unique reflections	82307
Completeness	91.8 (90.9)
Multiplicity	1.9 (1.9)
R_merge_	0.07 (0.47)
R_meas_	0.11 (0.88)
R_pim_	0.07 (0.60)
I/σ(I)	5.5 (1.1)
Refinement	
Resolution range (Å)	48.1-1.48
R/R_free_ (%)	18.8/21.4
RMSD bonds (Å)/angles (°)	0.007/1.143
Ramachandran plot, (% favoured/allowed/disallowed)	98.4/1.6/0
MOLPROBITY score/clash score	1.06/2.77

Values in parenthesis refer to the shell of highest resolution.

### Overall structure of azurin T21R1

**Figure 1:**
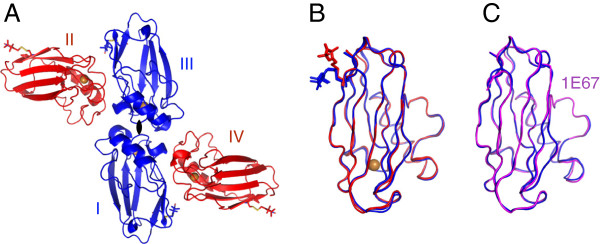
**Overall structure of triclinic azurin T21R1 crystals. A)** The asymmetric unit. The four azurin chains (I-IV) are depicted as cartoon models; the R1 side chains are shown as sticks. Brown spheres mark the positions of the copper ions. The two-fold NCS symmetry axis is indicated and the monomers are coloured to emphasize the NCS relationship. **B)** Superposition of all four monomers (colouring is identical to panel **A**). **C)** Superposition of monomer I with a previously published structure of azurin (magenta, PDB-ID: 1E67, [26]).

The asymmetric unit of spin labelled azurin T21R1 contains four independent monomers of azurin (I-IV) that are related by a two-fold NCS axis (Figure 1). The electron density for all four monomers is of excellent quality and a complete model with very good stereochemistry could be build (Molprobity score/clash score 1.06/2.77, Table 1). All four molecules have nearly identical structures amongst each other (RMSD = 0.3 Å for C_α_ atoms, Figure 1B) and to other azurin structures (e.g. RMSD = 0.31 Å for C_α_ atoms to 1E67 structure, Figure 1C).

### Structure of the R1 side chain

**Figure 2:**
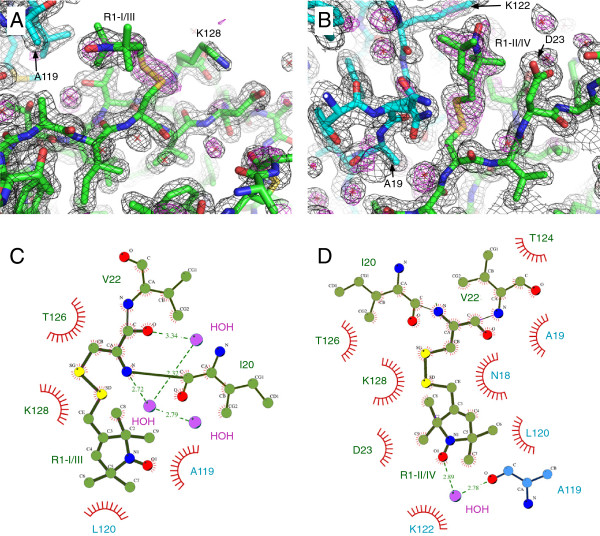
**Two different conformations of the R1 side chain. A)** The R1-I/III conformation. The structure of monomer I is shown as green sticks; the neighbouring azurin monomer is coloured blue. The black mesh is the refined 2mFo-DFc electron density contoured at 1 σ. The purple mesh is the positive difference electron density (mFo-DFc, contoured at 3.0 σ) that was observed before the spin label was added to the structural model during refinement. **B)** The R1-II/IV conformation. The figure is analog to panel **A**, but in this case the structure of monomer II is shown in green. **C)** Interaction topology diagram (Ligplot+, [29]) of the R1 side chain in monomer I (R1-I/III conformation) and its environment. Covalent bonds are indicated by solid lines, polar interactions by dashed lines and vdW interactions by red arcs. **D)** Same as panel C but for the R1 side chain in monomer II (R1-II/IV conformation).

After the molecular replacement step, positive difference electron density for the label was observed at the expected positions (T21). Following initial refinement with omitted R1 side chain, models of the label could be unambiguously placed into the electron density in all four monomers, resulting in two distinct conformations of the label (Figures 1B, 2A, B). In monomers I and III, the label is involved in vdW interactions with residues T126 and K128 of the same monomer and vdW interactions with residues A119 and L120 of a neighboring azurin monomer (Figure 2C). An analysis with the PISA server [28] revealed an accessible surface area of 228 Å^2^ for the R1 sidechain at this position, 49 Å^2^ of which are buried. Henceforth, the R1 conformation found in monomers I and III will be referred to as R1-I/III. In monomers II and IV, the R1 side chain lies at the heart of a crystal contact and is involved in numerous van-der-Waals (vdW) interactions and a hydrogen bond between the nitroxide oxygen and an ordered water molecule. This water molecule is again interacting with A119 of a neighboring azurin molecule (Figure 2B, D). For this R1 conformation (from here on referred to as R1-II/IV) PISA calculates a solvent accessible surface area of 260 Å^2^, 171 Å^2^ of which are buried. The higher number of stabilizing contacts in R1-II/IV is also reflected in a slightly higher quality of the electron density for this conformation (Figure 2A, B). Interestingly, a glycerol molecule from the cryo-protectant interacts with the main chain atoms of R1-II/IV (Additional file 1: Figure S1). In our structure, this molecule does not interact with the R1 side chain atoms. However, high concentrations of glycerol or ethylene glycole (up to 50%) are also used to cryo-protect PELDOR samples. Thus, it might well be, that in other cases, these molecules influence the dynamics of the spin label, either by direct interactions or indirectly by restricting its accessible volume.

### Mutual influence of R1 side chain and protein environment

**Figure 3:**
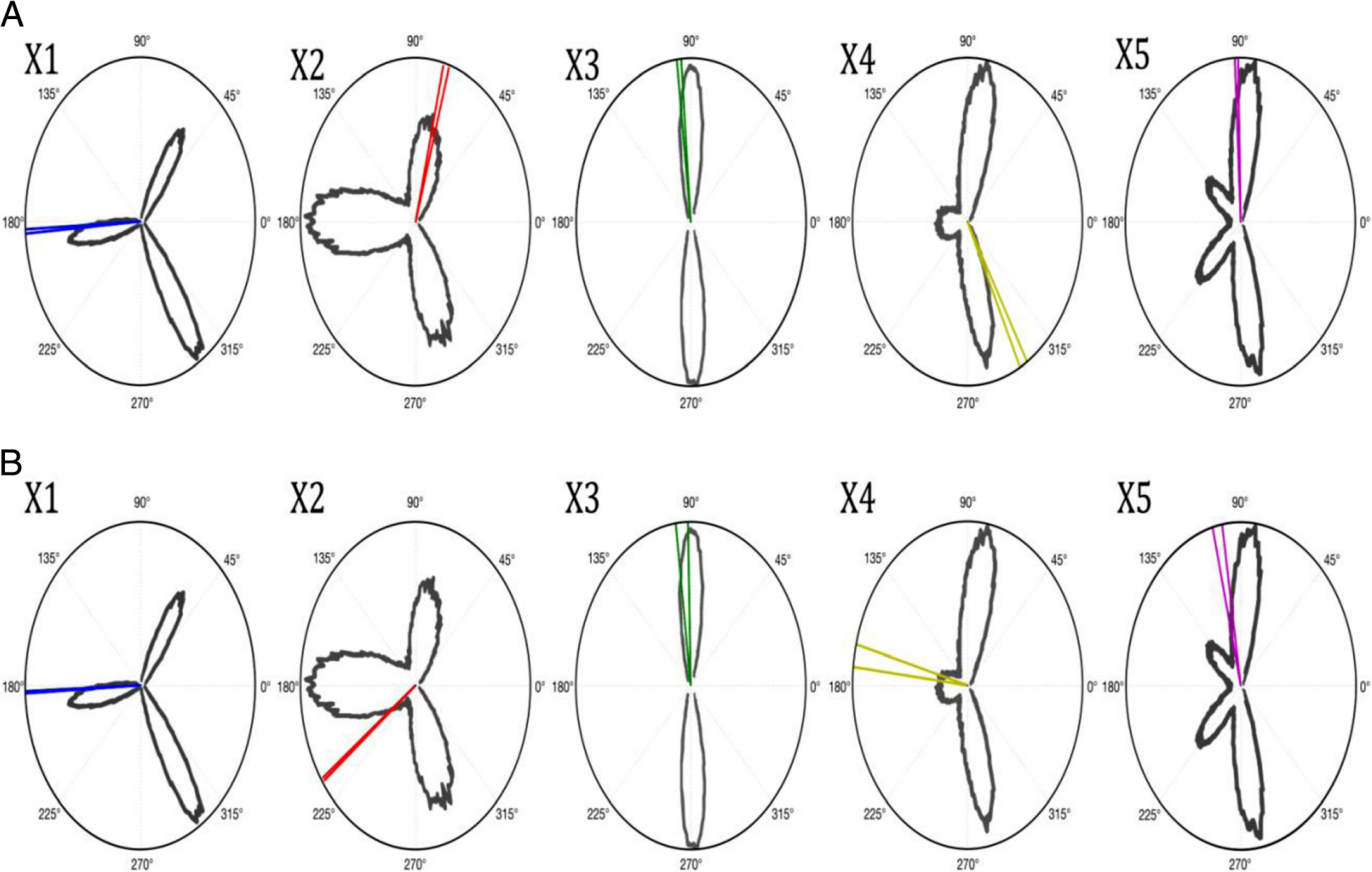
**Dihedral angles of the observed R1 side chains in azurin T21R1.** A polar plot is shown for each χ-angle. The angles measured in the R1-I/III conformation are plotted in **A** (coloured lines). The angles measured in the R1-II/IV conformation are plotted in **B** (coloured lines). The calculated angular distribution from the MTSSL rotamer library was digitized from [18] and is shown in each diagram (grey lines).

**Figure 4:**
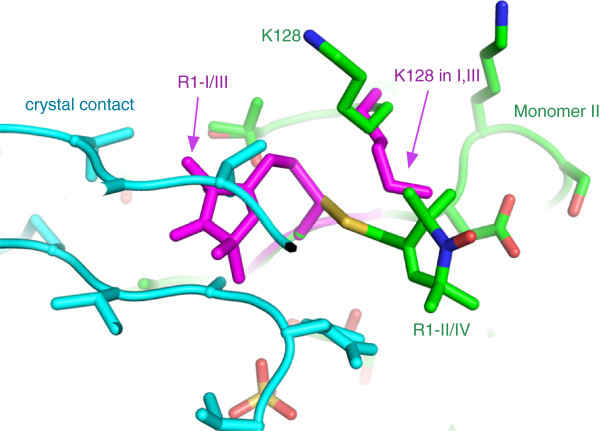
**The protein environment forces the R1 side chain into the R1-II/IV conformation.** A crystal contact of monomer II is shown in cyan. Side chains are represented as sticks. The alternative R1-I/III conformation of the R1 side-chain and the corresponding conformation of K128 are superposed (magenta).

Figure 3A shows polar plots of the dihedral angles (χ1- χ5) measured from the two R1 side chains in R1-I/III conformation. The two side chains were independently refined but have almost identical dihedral angles. The angles fit nicely to the angular distribution of a rotamer library derived from MD simulations of the free label [18]. In monomers II and IV, the crystal-packing environment precludes the R1-I/III conformation due to steric constraints (Figure 4). The label adjusts to this new environment and therefore adopts the R1-II/IV conformation. Again, the side chains were independently refined but show almost identical dihedral angles (Figure 3B). As described above, the R1-II/IV conformation is stabilised by multiple vdW interactions and a bridging water molecule (Figure 2D). Interestingly, the nearby K128 side chain rotates from the position found in monomers I and III by 180° to accommodate the R1-II/IV conformation (Figure 4). Figure 3B shows polar plots of the dihedral angles measured in the R1-II/IV conformation. Compared to R1-I/III, the χ_2_ and χ_4_ angles change significantly. At the same time, χ_5_ undergoes a smaller transition. Comparison with the rotamer library reveals that χ_2_, χ_4_ and to a lesser extend χ_5_ of R1-II/IV adjust to values that are less favorable for the free label (Figure 3B).

## Discussion

*In silico* spin labelling programs are important tools to translate EPR-derived distances into structural information, despite rather unsatisfying deviations between experimental and predicted distances [20,22,23]. To improve the algorithms, it is important to understand the reasons for these deviations, and to identify the most promising points for improvements. In published benchmark studies, the performance of individual programs is usually compared based on a comparison of predicted and experimental distances. But, the primary results of *in silico* spin labelling programs are predicted ensembles of spin labels. The two well-defined conformations of MTSSL in our crystal structure give rise to a number of inter-spin distances in the crystal packing and we thought this to be an interesting way to investigate how well predicted ensembles correlate with predicted distances. It should be noted that spin label conformations found in a crystal structure can be biased by interactions with the crystalline protein environment. However, in contrast to crystals of small molecules, protein crystals are interspersed by large solvent channels and usually consist of around 50% solvent. Also, before X-ray data are collected at low temperature, the crystals are typically cryo-protected by soaking them in e.g. 35% glycerol (even higher concentrations are used for cryo-protection in PELDOR samples). This prevents the formation of crystalline ice in the solvent channels, which would otherwise destroy the crystal or severly degrade its diffraction quality [30]. Thus, a spin label, which points into a solvent channel of a protein crystal is surrounded by the protein lattice, some ordered solvent molecules interacting with the spin-labelled protein and, a glassy, frozen solution of the solvent. Nevertheless, because of the flash-cooling process and interactions with the protein lattice, the spin label conformations observed in a crystal might be different from those that are found at room temperature and/or in liquid solution.

**Figure 5:**
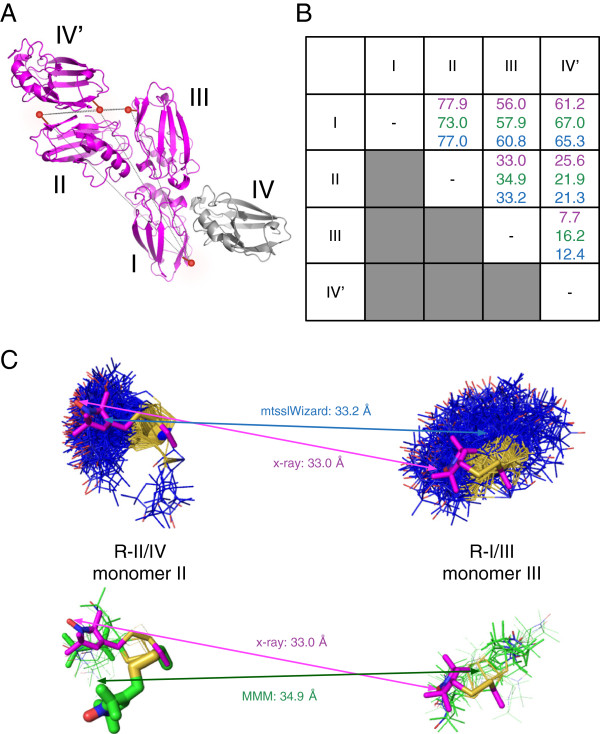
**Experimental and predicted distances between different azurin monomers in the azurin T21R1 crystal structure. A)** Selection from the crystal packing that was used as input for the spin labelling programs. Red spheres indicate the label positions. The grey monomer was included to complete the environment of the spin label on monomer I. **B)** Distances between the monomers in panel **A)**. Distances from the X-ray structure are pink, distances predicted by MMM are green and distances predicted by mtsslWizard are blue. For MMM the distances were taken from the .html output file generated by the program. **C)** close up of the II-III distance for mtsslWizard (top) and MMM (bottom). The experimental distance was taken from the X-ray structure and is shown in pink. The distance vector predicted by mtsslWizard is shown in blue and connects the geometric averages of the two ensembles predicted by the program (blue sticks). The green vector is the distance vector between the occupancy weighted geometric average of the MMM predicted ensembles (green sticks). For MMM, the occupancy of the individual rotamers is represented by the thickness of the sticks. Thus, only rotamers with a predicted occupancy larger than zero are shown.

To address the issue of interactions between label and protein lattice in our analysis, the complete subset of the crystal packing shown in Figure 5A was selected as input model for two programs: MMM (rotamer approach) and mtsslWizard (accessible volume approach). This selection contains the spin labelled monomers I-IV (Figure 5A) and, to complete the protein environment of each spin label, a symmetry equivalent of monomer IV (IV’) was additionally included (solvent molecules such as water and glycerol (see above) were excluded, because neither of the programs uses them). In principle, this selection can be seen as an experimental structure of one larger, continous protein chain (termed Azurin_BM_, as in benchmark) with multiple attached labels in either the R1-I/III- or R1-II/IV-conformation. Importantly, the complete protein environment of each label is present in this structure. For the purpose of the analysis below and for the reasons discussed above, we assume that in a flash frozen PELDOR sample of Azurin_BM_, the spin label will adopt very similar conformations to those that we found in the flash cooled azurin T21R1 crystals (Figure 2).

The table in Figure 5B compares the inter-label distances taken from this experimental structure to the equivalent distances obtained from mtsslWizard and MMM. Details of the MMM analysis (partition function, number of rotamers) are shown in Additional file 2: Figure S2. Further, a comparison of the predicted mean spin label positions is shown in Additional file 3: Figure S3. As found in larger distance-based benchmarks [20,22,23], both programs predict some of the distances quite accurately (e.g. II↔III), whereas large deviations are found for other distances (e.g. III↔IV’). This indicates that errors stemming from the generation of the spin label ensembles are sometimes compensated for by the relative geometric arrangement of a pair of ensembles. A similar observation has also been made in the crystal structure of the Spa15 chaperone [31]. Figure 5C illustrates this for the II-III distance: Whereas the absolute values of the distance vectors are very similar between X-ray structure, MMM and mtsslWizard, their directions differ considerably. This pair of labels is analysed in more detail below.

**Figure 6:**
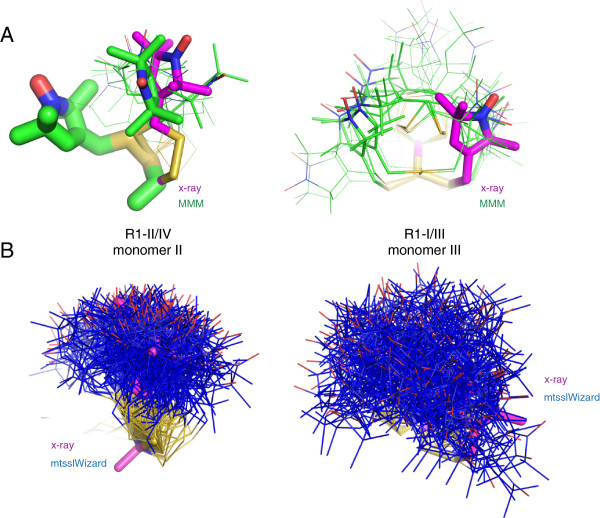
**The azurin T21R1 X-ray structure (MTSSL: pink sticks) in comparison with MTSSL rotamers predicted by MMM (A, green) and mtsslWizard (B, blue).** The predicted ensembles are shown as sticks and for MMM **(A)** the predicted occupancy is represented by the thickness of the sticks. Thus, only rotamers with a predicted occupancy larger than zero are shown.

The R1-I/III label in Figure 5C is located at a relatively “open” labelling site on monomer III (Figure 5A). In the crystal structure, the spin label at this site adopts a conformation that fits rather well to calculated rotamer libraries (Figure 3A). Figure 6A (right) shows a close-up of this site with the MTSSL ensemble predicted by MMM. Indeed, it contains some rotamers that are similar to the conformation found in the X-ray structure. But, even when only the rotamers from this ensemble are considered, the nitroxide group can still sample a large volume (Figure 6A, right). Thus, especially at open sites, it is crucial to also correctly predict the occupancy of the individual rotamers. In Figure 6A occupancies predicted by MMM are illustrated by the radius of the stick models [18]. In this case, the program outputs very similar occupancies for all rotamers. In contrast, the X-ray structure exhibits only one defined conformation and no indications for further conformations were found in the electron density (Figure 2A). Assuming, that due to its interactions with the environment (Figure 2A), this single conformation of the spin label would also be present in a PELDOR sample of Azurin_BM_, the failure to correctly predict the occupancy of the rotamers effectively neutralizes the advantage of the rotamer approach. This becomes clear by comparing the MMM ensemble with an ensemble predicted by mtsslWizard at the same site (Figure 6B, right). The overall volumes of both predicted ensembles are similar. Thereby, the uncertainty that will be introduced into predicted distances from or to this site will be similar between rotamer and accessible volume approach.

Clearly, the influence of the protein environment will be more pronounced at tight labelling sites, such as the one on monomer II (Figure 5C, left). As a result, deviations from the rotamer libraries become more likely, and we observe this in the crystal structure (Figure 3B). Consequentially, the R1-II/IV conformation cannot be correctly predicted when the rotamer library shown in Figure 3 is used (Figure 6A, left). It should be noted that this limitation of the rotamer approach at tight sites was pointed out by the authors of the MMM software [32]. In such cases, the rotamer approach effectively boils down to an accessible volume approach and would again deliver results that are very similar to the latter if the occupancies for all rotamers were set to the same value (Figure 6, left).

In the examples above, the spin labelled X-ray structure was used as a basis for *in silico* spin labelling. Usually, the problem is even more difficult, since it is unknown, how the protein will structurally react to the addition of the spin label. The K128 side chain in our structure is an example for a structural response of the protein (Figure 4). *In silico* spin labelling programs try to deal with this problem by allowing a certain number of clashes between protein and label. However, by doing this, the ensemble of created rotamers will simply grow in size whereas in reality the ensemble might merely change its shape, not necessarily its size. In the end this again leads to an increased uncertainty of the prediction.

## Conclusions

Our observations vividly illustrate why in our test case (and possibly also in general), the accessible volume approach and the more sophisticated rotamer approach often deliver very similar results. In essence, employing rotamer libraries will only increase the accuracy, when not only the rotamers but also their occupancy can be correctly predicted. The occupancy prediction is crucially dependent on the interaction of the label with its environment. The current software programs use only relatively simple (but fast) descriptions of the protein environment, whereas solvent molecules such as the bridging water shown in Figure 2 are completely ignored. Recently, the formation of hydrogen bonds has been found to be a very common type of interaction for nitroxide spin labels on proteins [33]. Also ignored are cryo protectants such as ethylene glycole or glycerol which are used at high percentages in PELDOR samples. These can bind close to the labelling site and thereby potentially influence the label dynamics (see above and Additional file 1: Figure S1). For labelling sites close to lipid bilayers or detergent micelles it is also important to consider label-lipid or label-detergent interactions since these will likely have profound effects on the conformation of the label. Thus, to increase the prediction accuracy, more sophisticated algorithms, which accurately account for label-environment interactions will have to be employed. It has been tried multiple times to use MD simulations for this purpose, but so far, the increased effort does not seem to pay off in terms of better prediction accuracy [34]. Promising ways to alleviate the described difficulties on the experimental side would be the use of spin labels with shorter or conformationally restrained linkers, such as the RX side chain [35] or the recently published V1 side chain [36].

## Methods

### Cloning, protein expression, purification and spin labelling

The gene for azurin (azu, PA4922) was PCR amplified from genomic *Pseudomonas aeruginosa* DNA using the PCR primers 5′-TTATAACCATGGCCGAGTGCTCGGTGG-3′ and 5′-CACCCTGACCCTGAAGTGAGAGCTCTTATAA-3′. The resulting PCR product did not contain the coding region for the N-terminal signal peptide of azurin (residues - 20 - 0), so that the target protein (residues 1 - 128 of azurin) could be expressed intracellularly in *E. coli*. The PCR product was then cloned into the vector pEHISGSTTEV (Huanting Liu, Biomedical Sciences Research Center, University of St Andrews, UK) via restriction enzymes NcoI and SacI, resulting in an expression construct with a TEV cleavable N-terminal His6-GST (glutathione S-transferase) tag. The T21C mutant was introduced into this construct using PCR. The resulting construct was transformed into *E. coli* Rosetta cells. A single colony was picked and grown over night in 50 ml of 2xYeast-Trypton (2YT) media supplemented with 100 μg/ml ampicillin and 17 μg/ml chloramphenicol with shaking at 37°C. On the following day, 1 l of 2YT medium with 50 μg/ml kanamycin and 17 μg/ml chloramphenicol were inoculated with 20 ml of the overnight culture and grown to an OD600 of 1.0. Protein expression was then induced by addition of 0.3 mM iso-propoyl-beta-thiogalactoside (IPTG). The protein expression was allowed to proceed for 3 h at 37°C with shaking at 200 rpm. The cells were then harvested by centrifugation at 2800 g, resuspended in 100 ml of lysis buffer (20 mM Tris-HCl pH 7.5, 500 mM NaCl, 30 mM Imidazol) and lysed with a cell disrupter at 30 kPsi (Constant Systems). Cell debris and insoluble proteins were spun down at 32.000 g for 15 min at 4°C. The soluble fraction was mixed with 1.5 ml Ni-NTA resin (Quiagen, pre-equilibrated in lysis buffer) and incubated for 1 h at 4°C with shaking. The resin was washed with 100 ml of lysis buffer, followed by 50 ml of lysis buffer supplemented with 1 mM tris(2-carboxyethyl)phosphine (TCEP) to reduce the introduced cysteine residue. The reducing agent was then quickly removed by washing the column with 50 ml of lysis buffer, directly followed by addition of 15 ml elution buffer (20 mM Tris-HCl pH 7.5, 500 mM NaCl, 1 M Imidazol) containing 0.7 mM of MTSSL. A large excess (~20×) of MTSSL was used, since the GST-tag of the expression construct also contained four cysteine residues. The labeling reaction was transferred to dialysis tubing and dialyzed over night against 5 l of dialysis buffer (20 mM Tris-HCl pH 7.5, 500 mM NaCl). On the next day, 4 mg TEV protease were added to the sample to cleave the GST-tag. The cleavage reaction was incubated for 3 h at room temperature. The sample was then concentrated to a volume of 2 ml, supplemented with 1 mM CuCl2 and loaded onto a Superdex200 16/60 column (GE) equilibrated with gel filtration buffer (10 mM Tris-HCl pH 8.0, 150 mM NaCl). Labelled monomeric azurin eluted at a volume of ~100 ml and had an intense blue colour.

### Crystallisation, data collection and refinement

Purified and MTSSL-labelled azurin T21R1 was concentrated to 10 mg/ml for crystallization and sitting drop crystallization setups were prepared with the commercial JCSG + screen (Molecular Dimensions) in MRC plates (Molecular Dimensions). Blue, plate shaped azurin crystals grew within 2-3 days at room temperature in conditions A2, B7, C4 and C11. The components of these conditions where then used for a stochastic optimization and led to the final condition: 2.14 M ammonium sulfate, 0.28 M ammonium nitrate, 0.1 M sodium cacodylate pH 6.0. For data collection, the crystals were harvested and cryo-protected with 35% glycerol prior to flash cooling in liquid nitrogen. A diffraction data set was collected at BESSYII, BL14.1 (Berlin) using a PILATUS 6M detector. The data were indexed (iMOSFLM [37]) in space group P1 and processed with iMOSFLM, POINTLESS and SCALA [38]. The structure of Zn^2+^-bound azurin (PDB-ID: 1E67) was used as model for molecular replacement with PHASER [39]. The program located all four azurin molecules in the asymmetric unit. The monomers (I-IV) are related by a two-fold non-crystallographic symmetry between I, II and III, IV. The model was refined automatically using PHENIX.REFINE [40] and by hand using COOT [41]. Data collection and refinement statistics are listed in Table 1.

## Competing interests

The authors declare that they have no competing interests.

## Authors’ contributions

NF & GH performed research. GH and OS designed research. GH and OS wrote the paper. All authors read and approved the final manuscript.

## Supplementary Material

Additional file 1: Figure S1 A glycerol molecule (lilac) from the cryo protectant interacts (dotted lines) with the main chain atoms of the R1 label. The protein structure is shown as green sticks, the neighbouring protein in the crystal is shown as blue sticks. Water molecules are shown as red crosses. Click here for file

Additional file 2: Figure S2 Cartoon image of the structure that was used as input for the in silico spin labelling programs. The colouring is identical to Figure 5 but the partition functions (p.f.) and number of rotamers (#) generated by MMM are given. The label on monomer IV was ignored because its complete protein environment is not part of the ensemble. Click here for file

Additional file 3: Figure S3 Cartoon image of the structure that was used as input for the in silico spin labelling programs. The colouring is identical to Figure 5. The average coordinates from MMM (green spheres), mtsslWizard (blue spheres) and the N1 coordinates from the X-ray structure are shown. Click here for file
