# Supplementary material for: High-resolution crystal structure of spin labelled (T21R1) azurin from Pseudomonas aeruginosa: a challenging structural benchmark for in silico spin labelling algorithms
Source: BMC Struct Biol. 2014 May 29;14:16. doi: 10.1186/1472-6807-14-16 (PMC4055355; doi:10.1186/1472-6807-14-16)
Supplement: Additional file 1: Figure S1 — A glycerol molecule (lilac) from the cryo protectant interacts (dotted lines) with the main chain atoms of the R1 label. The protein structure is shown as green sticks, the neighbouring protein in the crystal is shown as blue sticks. Water molecules are shown as red crosses. [file 1472-6807-14-16-S1.pdf]

Supplementary Figure 1

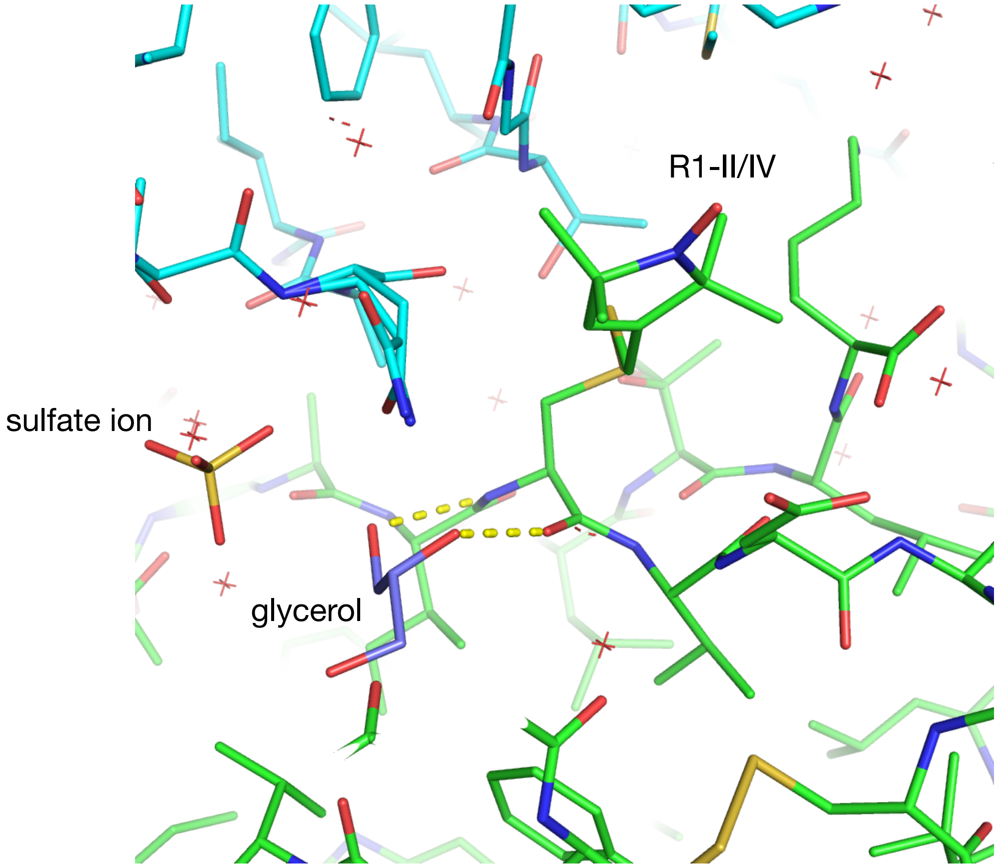

Supplementary Figure 1: A glycerol molecule (lilac) from the cryo protectant interacts (dotted lines) with the main chain atoms of the R1 label. The protein structure is shown as green sticks, the neighbouring protein in the crystal is shown as blue sticks. Water molecules are shown as red crosses.
