# Supplementary material for: High-resolution crystal structure of spin labelled (T21R1) azurin from Pseudomonas aeruginosa: a challenging structural benchmark for in silico spin labelling algorithms
Source: BMC Struct Biol. 2014 May 29;14:16. doi: 10.1186/1472-6807-14-16 (PMC4055355; doi:10.1186/1472-6807-14-16)
Supplement: Additional file 2: Figure S2 — Cartoon image of the structure that was used as input for the in silico spin labelling programs. The colouring is identical to Figure 5 but the partition functions (p.f.) and number of rotamers (#) generated by MMM are given. The label on monomer IV was ignored because its complete protein environment is not part of the ensemble. [file 1472-6807-14-16-S2.pdf]

Supplementary Figure 2

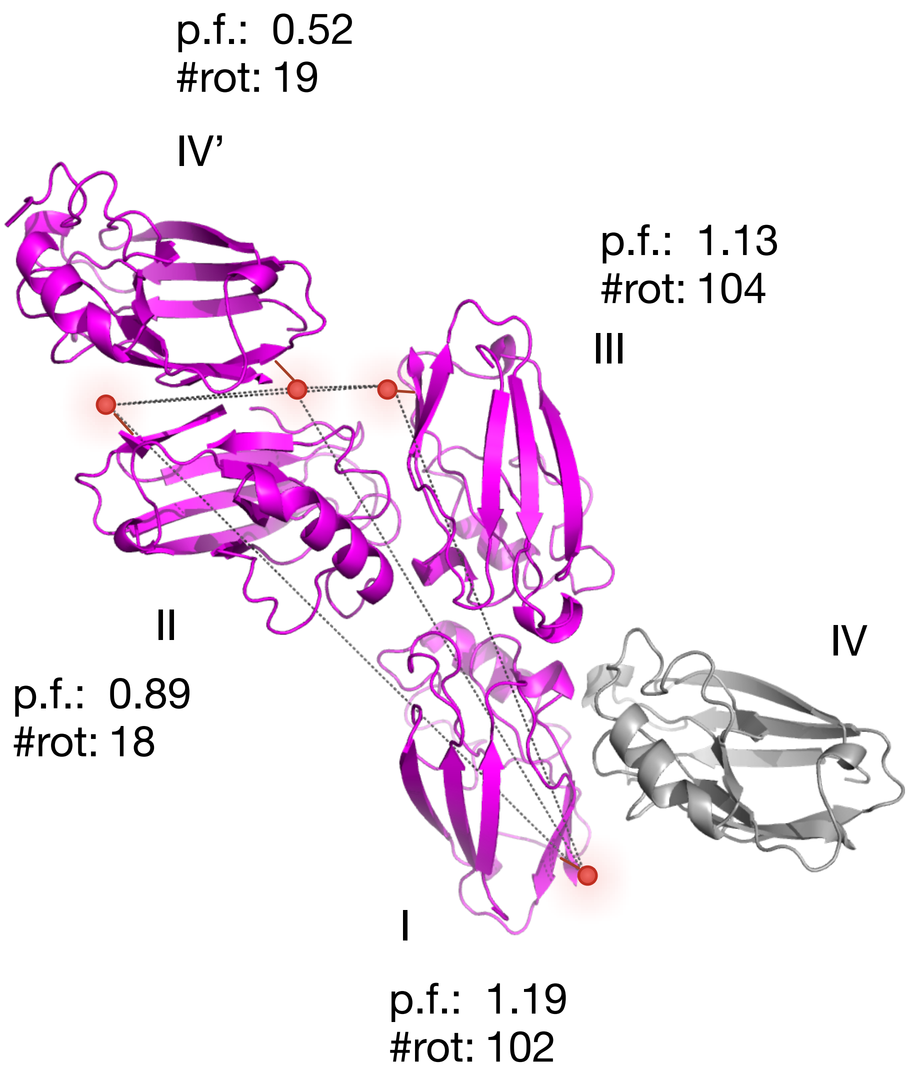

Supplementary Figure 2: Cartoon image of the structure that was used as input for the in silico spin labelling programs. The colouring is identical to Figure 5 but the partition functions (p.f.) and number of rotamers (#) generated by MMM are given. The label on monomer IV was ignored because its complete protein environment is not part of the ensemble.
