# Supplementary material for: High-resolution crystal structure of spin labelled (T21R1) azurin from Pseudomonas aeruginosa: a challenging structural benchmark for in silico spin labelling algorithms
Source: BMC Struct Biol. 2014 May 29;14:16. doi: 10.1186/1472-6807-14-16 (PMC4055355; doi:10.1186/1472-6807-14-16)
Supplement: Additional file 3: Figure S3 — Cartoon image of the structure that was used as input for the in silico spin labelling programs. The colouring is identical to Figure 5. The average coordinates from MMM (green spheres), mtsslWizard (blue spheres) and the N1 coordinates from the X-ray structure are shown. [file 1472-6807-14-16-S3.pdf]

Supplementary Figure 3

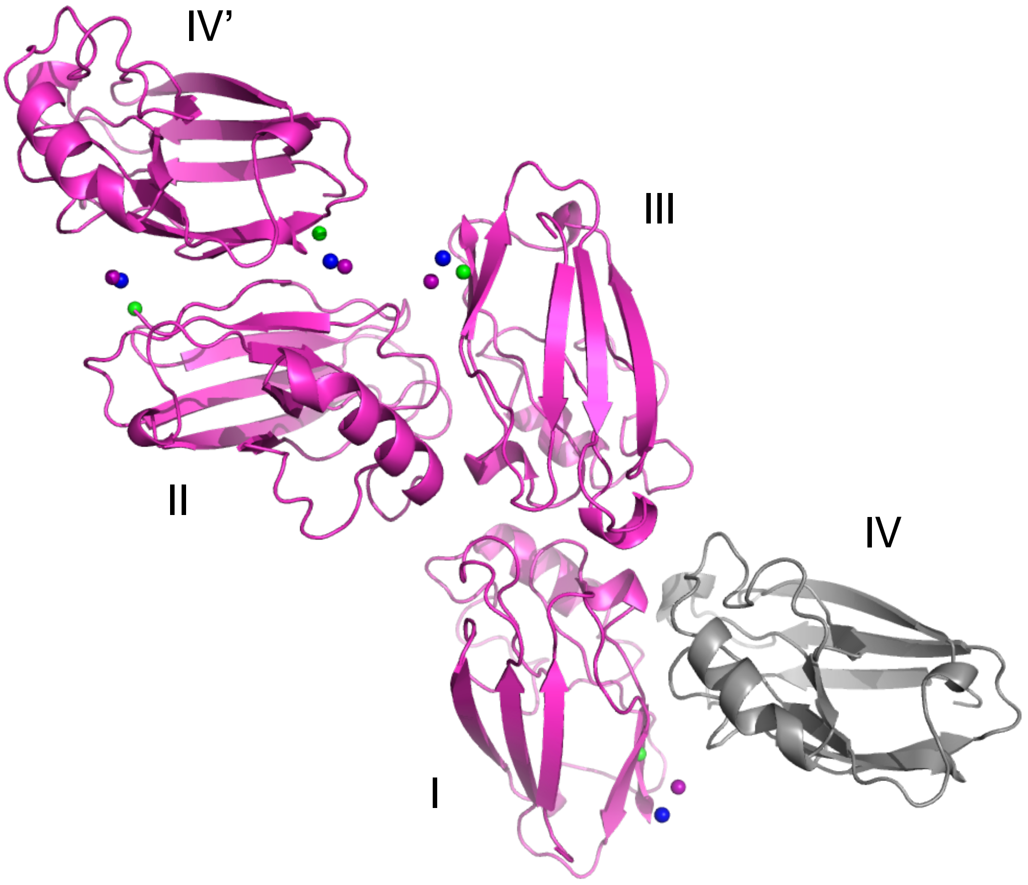

Supplementary Figure 3: Cartoon image of the structure that was used as input for the in silico spin labelling programs. The colouring is identical to Figure 5. The average coordinates from MMM (green spheres), mtsslWizard (blue spheres) and the N1 coordinates from the X-ray structure are shown.
